# Insanity in Egypt

**Published:** 1847-01

**Authors:** 


*Insanity in Eygpt.*—The following extract from a clever work just published, conveys a wretched picture of the condition of the insane in Egypt. It is to be hoped that Ibrahim Pacha was not allowed to leave this country without witnessing the treatment pursued at Hanwell and others of our best conducted asylums.

"The saddest sight I have seen in Cairo is the Mooristan or madhouse—misery mitigated by nothing but its own oblivious antidote—'Razing the written troubles of the brain.'

"A horrid court-yard, surrounded by tiers of iron cages, where men are cooped, and sometimes chained, with less of space, air, light, and cleanliness, than are allowed to a wild beast in one of our travelling menageries. Poor helpless wretches!—the moping idiot, the gay madman, the furious maniac,—sullen and weeping, laughing and singing, grinning, howling, and tearing behind the bars;—of all the fearful 'ills that flesh is heir to,' this overthrow of reason is surely the most painful to look upon.

. . . . . . . . omni
'Membrorum damno major Dementia.'

I saw one poor patient brought out of his den and set at liberty; he lay for a few minutes upon a filthy mat on the stone pavement, his features drawn, lived, and stiff—a shudder passed through his wasted frame, and he was dead!

"After several visits, I have established an acquaintance with two or three, and make them presents of bread and tobacco, for which they are very eager. One captive, quiet, self-collected, and hand some featured, tells a long well-sustained story of female jealousy and a family conspiracy to obtain his property and confine him for life : he stoutly maintains that he is as much in his senses as any man in Muzr—a strong reason for doubting the tale, which if true, would have driven a philosopher crazy. My new servant, *Black Omar*, who stands interpreter, believes every word, and thinks it no uncommon case ; he tells me that *snake broth* is the 'sovereignis't thing on earth' for these mental maladies, and that no other medicine is used in the Mooristan, save the iron-chained collar and the bastinado,"—*Nozrani in Egypt and Syria.*

